# Non-destructive detection of internal egg defects: Transmission imaging for blood spots and Vis-NIR spectroscopy for runny yolk

**DOI:** 10.1016/j.psj.2026.107220

**Published:** 2026-06-02

**Authors:** Abdullah Al-Mamun, Md. Abdullah Al Noman, Iqbal Hossain, Jannatul Ferdushi Jany, Md. Anisur Rahman Mazumder, Abdullah Iqbal, Mohammad Gulzarul Aziz, Afzal Rahman

**Affiliations:** Department of Food Engineering and Technology, Faculty of Agricultural Engineering and Technology, Bangladesh Agricultural University, Mymensingh 2202, Bangladesh

**Keywords:** Spectroscopy, Egg, Chemometrics, Machine learning

## Abstract

Ensuring the internal quality of eggs is essential for food safety and industrial-scale grading. While current systems can detect blood spots, non-destructive identification of runny yolk remains a major challenge. This study evaluates a parallel, non-destructive approach utilizing transmission imaging specifically for blood spot identification, alongside visible-near-infrared (Vis-NIR) spectroscopy for the detection of both blood spots and runny yolk. In the imaging system, blood spots were detected based on pixel area thresholds, achieving up to 92.00% accuracy in white eggs and 88.00% in brown eggs. Using supervised classification, Linear Discriminant Analysis (LDA) yielded the best performance among image-based methods. However, Vis-NIR spectroscopy outperformed imaging, with Quadratic Discriminant Analysis (QDA) achieving 97.50% and 98.37% accuracy for blood spot detection in white and brown eggs, respectively. Notably, this study reports the first successful non-invasive detection of runny yolk, reaching 99.01% accuracy in white eggs and 100.00% in brown eggs using LDA. This was enabled by identifying distinct lipid absorption features in the 500- 600 nm range. Although induced defects were used for validation, the results demonstrate the feasibility of a robust, scalable framework for intelligent egg quality assessment, advancing automation in the poultry industry.

## Introduction

Chicken eggs are a globally consumed food, valued for their high nutritional content, including proteins, vitamins, and omega-3 fatty acids (Nys et al., 2011). Egg quality, encompassing consumer preference and safety, is categorized into two categories. External quality (e.g., shell cleanliness, soundness, and size) is easily assessed and is a primary driver of consumer choice (Jibir et al., 2012). Conversely, internal quality, which involves the albumen and yolk conditions, is more difficult to evaluate non-destructively. Internal metrics, such as the Haugh unit and albumen height, are highly correlated with each other and show a moderate link to external traits, such as shell color (Jang, 2022).

Internal defects, such as blood spots and runny yolk, significantly degrade egg quality. Blood spots, caused by hemorrhages during ovulation, can affect 2-10% of eggs and negatively impact consumer purchasing decisions . Runny yolk, characterized by the breakdown of the vitelline membrane and leakage of yolk into the albumen, is another serious defect. This can be caused by factors such as the age of the hen, storage conditions, and the handling procedures. In the food industry, particularly in bakeries where egg whites are used for their foaming properties, even trace amounts of yolk contamination (as little as 0.05%) can drastically impair functionality (Cook and Mehlenbacher, 1946; G. Wang and Wang, 2009).

Current industrial standards for egg internal quality inspection primarily use candling- either manual visual inspection against bright light or automated systems to detect shell cracks and internal features like air cell size, albumen clarity, and yolk position. These methods effectively identify visible shell cracks but struggle with subtle internal defects (So et al., 2022).

Manual candling remains labor-intensive, requiring skilled operators to examine eggs individually in darkened environments, often leading to tedium and health issues like eye strain or headaches from prolonged dark-room work. The process is subjective, with consistency varying by operator experience, and prone to errors such as missing 10-20% of cracks in high-speed production (6 eggs/second per operator) (Vargas Cruz Ramiro Sebastián et al., 2018).

Detection accuracies for internal defects and fertility often fall below 80%, particularly influenced by operator fatigue; for example, manual candling of 5-7 days incubated duck eggs achieves only 40-70% correct detection. Automated systems improve speed and consistency (e.g., 86.00% accuracy in some prototypes) but may miss nuanced defects humans catch, and overall rates for complex internal issues remain variable below 90-95% in studies. Traditional methods' limitations highlight the need for advanced nondestructive technologies in modern production (Garcia and Magwili, 2021).

In response to these limitations, various automated, non-destructive techniques have been explored, including machine vision and spectroscopy (Garcia-Alegre et al., 2000; Usui et al., 2003). Machine vision has been applied to detect shell defects and blood spots (V. C. Patel et al., 1996; Pourreza et al., 2008), and spectroscopy has shown promise for assessing freshness and internal properties (Chen et al., 2015; Kemps et al., 2006), these methods have limitations when used in isolation.

To date, a reliable, non-destructive method for detecting runny yolk has not been reported. Furthermore, the combined application of imaging and Vis-NIR spectroscopy for detecting both blood spots and runny yolk in white and brown eggs has not been studied extensively. Unlike previous research that focuses on algorithmic development for existing defect categories, this study addresses this critical gap by developing and evaluating a system that uses transmission imaging and Vis-NIR spectroscopy parallelly with multivariate analysis. We hypothesized that the distinct biochemical changes associated with blood spots (presence of hemoglobin) and runny yolk (diffusion of lipids) would produce unique optical signatures that could be accurately classified. The primary objective of this study was to establish a highly accurate and scalable method for the non-destructive detection of these internal defects, paving the way for advanced automated quality grading systems in the poultry industry.

## Materials and methods

### Sample preparation

Fresh, unwashed table eggs were sourced from 45-week-old White Leghorn (white-shelled) and Novogen Brown (brown-shelled) laying hens maintained on a standard diet at the Bangladesh Agricultural University Poultry Farm and a commercial hatchery in Mymensingh, Bangladesh. All experimental procedures were conducted at the Department of Food Engineering and Technology, Bangladesh Agricultural University, in accordance with institutional ethical standards.

A total of 800 fresh table eggs were used in this study to ensure a balanced dataset for classification. For the blood spot detection experiment, 400 eggs were utilized: 200 white eggs (100 control, 100 artificially induced blood spots) and 200 brown eggs (100 control, 100 blood spots). Similarly, for the runny yolk detection experiment, a separate set of 400 eggs was prepared, comprising 200 white eggs (100 control, 100 runny yolk) and 200 brown eggs (100 control, 100 with the runny yolk defect). This sample size provided 100 replicates per class, ensuring sufficient statistical power for training and validating the machine learning models.

Internal defects were artificially induced to establish a standardized dataset for subsequent model development. For blood spot simulation, a 0.3 mm diameter hole was drilled in the eggshell through which fresh chicken blood was injected. The injection volume was 0.02 mL for white-shelled eggs and increased to 0.04 mL for brown-shelled eggs to compensate for the greater light absorbance resulting from higher shell pigmentation. These specific volumes were selected to simulate moderate-to-large blood spots, which are strictly classified as rejectable defects under commercial grading standards. Furthermore, these fixed volumes were determined through empirical preliminary trials designed to guarantee ground-truth detectability. In white eggs, 0.02 ml provided a reliable hemoglobin absorption signature. However, the presence of protoporphyrin IX pigment in brown shells significantly attenuates light transmission. Preliminary testing revealed that a 0.04 ml volume was the empirical minimum required to overcome this baseline pigment attenuation and ensure a localized optical contrast (Contrast-to-Noise Ratio) comparable to that achieved in the white eggs.

To simulate a runny yolk, a similar 0.3 mm aperture was created, and a 0.2 mm diameter metal wire was inserted and rotated ten times to mechanically rupture the vitelline membrane, inducing yolk dispersal into the albumen.

Fig. 1.

**Fig. 1:**
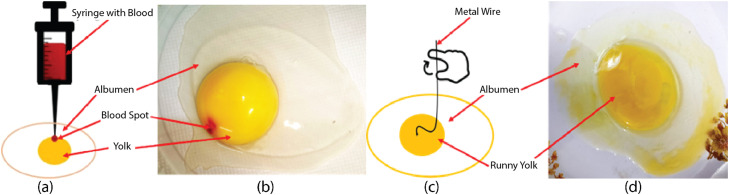
Illustration of the methods for inducing artificial internal egg defects. (a) Schematic of the blood injection process and (b) the resulting blood spot. (c) Schematic showing the mechanical rupture of the Vitelline Membrane and (d) the subsequent Runny Yolk.

### Instrumentation and data acquisition

#### Transmission image acquisition

**Fig. 2:**
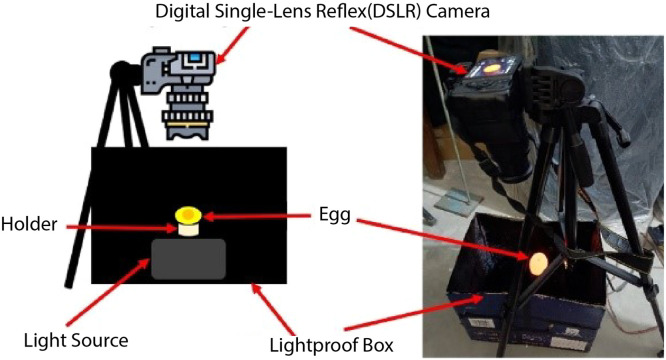
Schematic (a) diagrams and (b) pictorial of the transmission image acquisition system.

An image acquisition system was constructed to capture the light transmitted through the eggs. The system consisted of a lightproof box, a 50 W halogen lamp (OSRAM HALOSTAR 64440) as a light source, and a DSLR camera (Nikon D3400) with an 18–55 mm lens. To ensure consistency, the camera was calibrated with a fixed F-number (f/5.6), focal length (55 mm), ISO (2800), and shutter speed (1/40 s). The images were saved in JPEG format for processing. While manual dark-current and flat-field calibrations were not explicitly applied to raw sensor data, the camera's internal processing engine applies baseline noise reduction and lens profile corrections. Furthermore, geometric distortion was mitigated by positioning the egg centrally on the optical axis within the controlled lightproof box. The minor illumination heterogeneities inherent in this setup were successfully accommodated by the robust nature of the subsequent Otsu global thresholding algorithm. However, transitioning this system to an industrial environment utilizing uncompressed machine vision cameras will necessitate the implementation of standard flat-field and dark-current calibrations to ensure continuous reproducibility. Fig. 2.

#### Spectral acquisition

A portable Vis-NIR spectrophotometer (LinkSquare, Stratio Inc., USA) was used to measure the transmission spectrum of each egg over the range of 400–1000 nm. The eggs were placed on a custom-designed 3D-printed holder above the 50 W halogen lamp within a lightproof box. This holder ensured a consistent vertical orientation for all samples and maintained a fixed source-to-sample distance of 5 mm. Ten spectra were collected for each egg and averaged. Preliminary analysis revealed that light transmittance below 500 nm was negligible due to the high absorbance of the eggshell and membrane, resulting in a low signal-to-noise ratio. Consequently, the spectral analysis was restricted to the 500–1000 nm range to ensure data reliability. The raw transmission spectra (X_raw_) were corrected for electrical noise (X_noise_) and transformed into proportional transmission (X) using a reference spectrum (X_ref_) with the following equation (Kemps et al., 2006): Fig. 3.

(1) $$Xi=\frac{Xraw-Xnoise,i}{Xref,i-Xnoise,i}$$

**Fig. 3:**
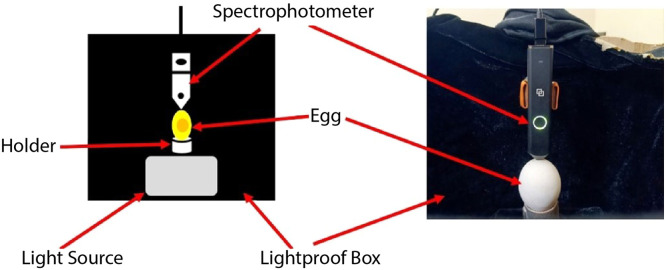
Schematic (a) and pictorial (b) diagrams of the transmission Spectral Acquisition System.

### Data analysis

#### Image processing for blood spot detection and runny yolk detection

**Fig. 4:**
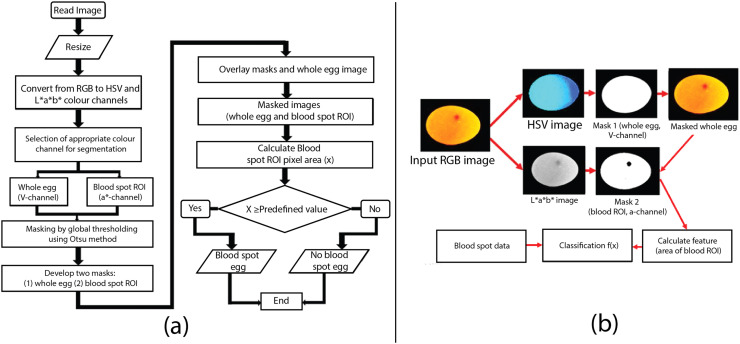
Image processing workflow for blood spot detection, showing (a) a detailed process flowchart and (b) a schematic outline of the masking and classification steps.

The image processing workflow for blood spot detection, illustrated in Fig. 4, began with preprocessing, where each image was resized to standardize the dimensions and then converted from the RGB color space to both the HSV (Hue, Saturation, Value) and L*a*b* color spaces. This conversion was performed to identify the most effective channels for segmenting the egg and defects. The V-channel was selected for segmenting the entire egg from the background. For blood spot isolation, the a-channel (representing the green-red color opponent) was determined to be the optimal choice. This is physically justified because hemoglobin exhibits a strong absorption peak in the green region of the spectrum (approximately 540–580 nm) (Kim et al., 2022). Consequently, blood spots appear significantly darker than the surrounding yolk and albumen in the a-channel, providing the highest contrast-to-noise ratio compared to the HSV or RGB channels, which facilitated accurate thresholding. Segmentation was achieved using the Otsu global thresholding method, which algorithmically determines an optimal threshold to separate pixels into two classes by minimizing the intra-class variance (Otsu, 1979). This method, consistent with previous studies on egg defect detection (Li et al., 2012; V. C. Patel et al., 1996), was applied to the selected channels to create two binary masks: one for the entire egg and one for the blood spot Region of Interest (ROI). The area of the blood spot ROI (in pixels) was then calculated and used as the primary feature for the classification. An egg was classified as defective if its blood spot ROI pixel area (x) exceeded the predefined threshold (β=80). This specific threshold value was not arbitrary; it was derived empirically through an iterative search applied exclusively to the training subset. By evaluating threshold values from 10 to 200 pixels, β = 80 was identified as the global optimum that maximized the F1-score, effectively balancing the detection of true blood spots against false positives caused by natural chalazae or shell variations. While this global threshold proved highly effective overall, its applicability varies slightly by shell type. The threshold yielded a 92.00% detection accuracy for white eggs but 88.00% for brown eggs. This discrepancy indicates that the higher melanin content in brown shells reduces the optical contrast within the a-channel, making the defect boundary less distinct. Therefore, while a static threshold of 80 is computationally efficient, future industrial applications would likely benefit from a dynamic, color-adaptive thresholding algorithm to maximize detection accuracy across varying degrees of shell pigmentation.

The same image processing pipeline was applied to eggs with artificially induced, runny yolk.

Although the thresholding method successfully segmented the entire egg from the background, it completely failed to identify a Region of Interest (ROI) for the runny yolk defect. To quantitatively evaluate this failure, the Contrast-to-Noise Ratio (CNR) between the localized runny yolk diffusion and the surrounding albumen was calculated across the HSV and L*, a*, b* color spaces. For reliable image segmentation, a CNR of 2.0 is typically required. Our analysis revealed that the CNR for the runny yolk defect was 1.0 in all evaluated channels. This negligible contrast value mathematically demonstrates that the pixel intensity of the diffused yolk lipids overlaps almost entirely with the natural variance of the albumen. Because the threshold distributions for the defect and the background are practically indistinguishable, image-based segmentation is quantitatively unviable for this specific defect, underscoring the necessity of the spectroscopic approach.

While the algorithm successfully segmented the entire egg from the background, it was unable to isolate a Region of Interest (ROI) corresponding to the runny yolk. This limitation is fundamentally optical rather than algorithmic. Unlike blood spots, which possess distinct boundaries and high localized absorption, a runny yolk represents the chemical diffusion of lipids and proteins into the albumen (Abeyrathne et al., 2022). Under visible transmission, this diffusion creates a continuous, low-frequency spatial gradient rather than a definable edge. Because the light attenuation of the diffused yolk closely mirrors the natural variations in albumen thickness, the optical Contrast-to-Noise Ratio (CNR) approaches zero in the captured color channels (Ahmed et al., 2025). Consequently, advanced spatial image analysis techniques- including edge detection or deep learning semantic segmentation- are unlikely to succeed, as the required morphological boundaries and pixel contrast are physically absent in the transmission images. This fundamental lack of spatial contrast underscores the necessity of the spectroscopic approach, which bypasses morphological limitations to directly detect the biochemical signature of the diffused lipids.

#### Spectral data preprocessing and chemometric analysis

##### Preprocessing and feature selection for blood spot detection

**Fig. 5:**
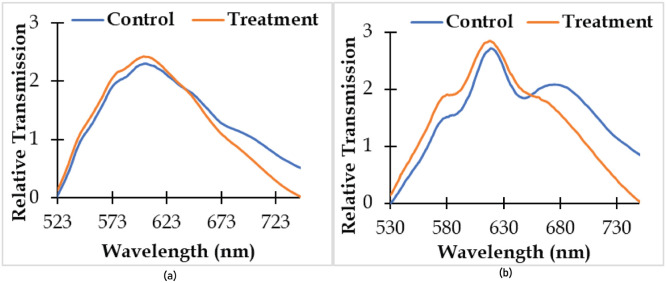
Average transmission spectra of (a) white eggs and (b) brown eggs for blood spot detection.

The corrected transmission spectra were imported into the ‘Unscrambler X’ software for chemometric analysis. Raw spectral data can be affected by physical variations between samples, such as egg size and shell thickness, and instrumental noise. To address these issues, a series of preprocessing steps was applied. While derivative treatments were considered, SNV and smoothing were intentionally selected to normalize the spectra and correct multiplicative interferences from light scattering without amplifying the high-frequency instrumental noise inherent in low-intensity transmission setups. Furthermore, Principal Component Analysis (PCA) was deliberately chosen as an unsupervised feature extraction tool. Utilizing unsupervised PCA loadings ensured that the identified spectral variables naturally aligned with the underlying biochemical changes, mitigating the risk of overfitting that supervised feature selection algorithms might introduce on an artificially induced dataset. Standard Normal Variate (SNV) transformation was used to normalize each spectrum, effectively correcting for multiplicative interferences from light scattering and path length variations inherently caused by physical differences between individual samples, such as natural fluctuations in egg weight, shell thickness, and air cell size (Kemps et al., 2006). Following the SNV, a smoothing filter was applied to reduce the high-frequency instrumental noise and enhance the underlying spectral features. Owing to the low light intensity of the halogen lamp in the near-infrared region and low light transmittance through the eggshell below 500 nm, the spectral analysis was focused on the 500–750 nm range. The average preprocessed transmission spectra for the blood spots and control eggs are shown in Fig. 5. A clear difference can be observed, with the blood spot eggs exhibiting lower relative transmission (indicating higher absorbance) in the 540-580 nm region, which is consistent with the known ‘Soret Absorption Bands of Hemoglobin’ (Rahman et al., 2020).

**Fig. 6:**
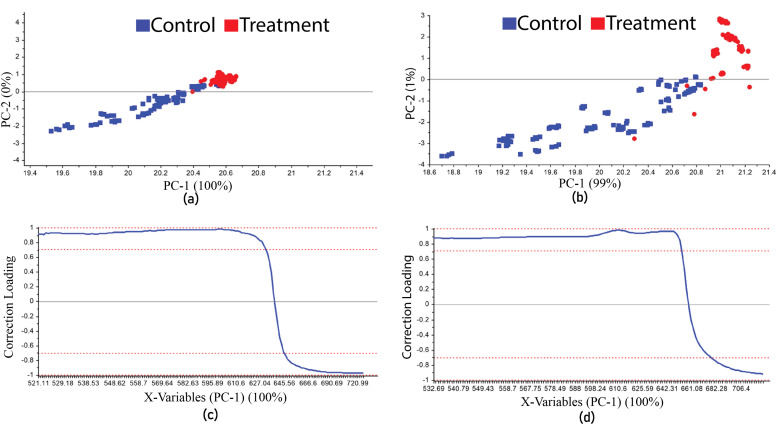
Principal component analysis (PCA) of spectral data for blood spot detection. (a) PC-1 vs. PC-2 score plot for white eggs. (b) PC-1 vs. PC-2 score plot for brown eggs. (c) Correlation loading plot for PC-1 of white eggs. (d) Correlation loading plot for brown eggs.

Principal Component Analysis (PCA) was subsequently used as an exploratory data analysis tool to reduce the dimensionality of the spectral data and identify the most influential wavelengths. PCA decomposes the spectral matrix into a set of orthogonal variables called principal components (PCs), where the first few PCs explain most of the variance in the data. Score plots of the first two PCs (PC-1 vs. PC-2) were generated to visualize the data structures. As shown in Fig. 6 (a and b), these plots revealed clear clustering and separation between the control (no defect) and treatment (blood spot) egg groups, confirming that the primary sources of variation captured by the model were related to the presence of the defect. For both white and brown eggs, the separation occurred almost entirely along the PC-1 axis, which accounted for 100.00% and 99.00% of the variance, respectively. This indicates that PC-1 effectively represents the spectral signature of blood spots.

Correlation loading plots for PC-1 were analyzed to identify the specific wavelengths responsible for this separation, as shown in Fig. 6 (c and d). These plots show the correlation between each original wavelength and the principal components. Wavelengths with high absolute loading values (close to +1 or −1) were the most significant contributors to the observed separation. For white eggs, the influential wavelengths were identified in the ranges of 521–635 nm and 645–730 nm. For brown eggs, the key ranges were 532–653 nm and 682–732 nm, respectively. This feature selection step allowed for the narrowing of the spectral data to only the most informative variables, which were then used as inputs to build more robust and accurate classification models with fewer features.

##### Preprocessing and feature selection for runny yolk detection

**Fig. 7:**
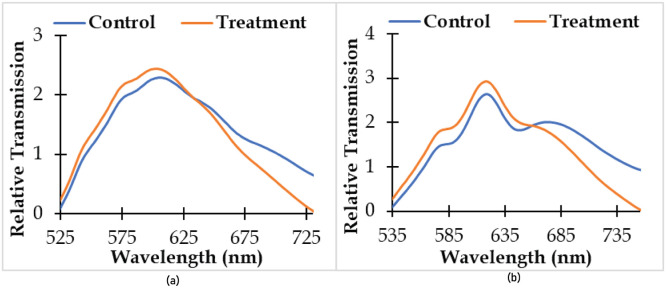
Average transmission spectra of (a) white and (b) brown eggs for Runny Yolk detection.

The same preprocessing steps (SNV and smoothing) were applied to the spectral data from the runny-yolk experiment. The average spectra (Fig. 7) show a lower overall transmission profile for eggs with runny yolk than for the control group. This is attributed to the increased light scattering and absorption caused by the diffusion of yolk components, such as lipids and proteins, into the albumen (Pickering, 1992).

**Fig. 8:**
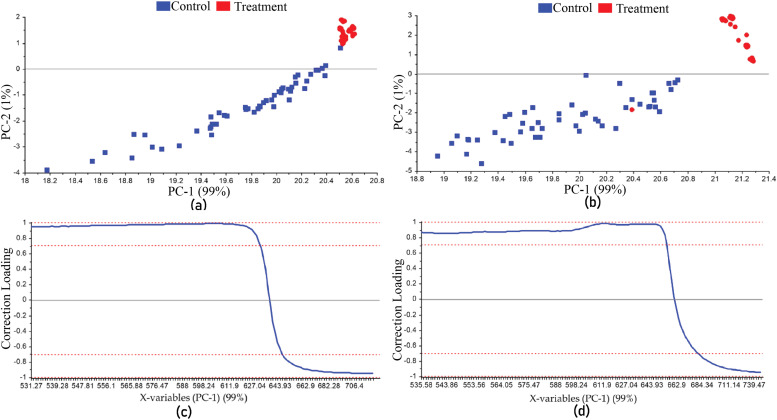
Principal component analysis (PCA) of spectral data for Runny Yolk detection. (a) PC-1 vs. PC-2 score plot for white eggs. (b) PC-1 vs. PC-2 score plot for brown eggs. (c) Correlation loading plot for PC-1 of white eggs. (d) Correlation loading plot for brown eggs.

Principal Component Analysis (PCA) was performed to explore the data structure and identify the key wavelengths. The PCA score plots for runny yolk detection are shown in Fig. 8 (a and b). For both white and brown eggs, a distinct separation was evident between the control and treatment groups, primarily along the PC-1 axis, which explained 99.00% of the total variance in both cases. This strong separation indicates that the spectral changes caused by yolk diffusion are the dominant source of variation captured by the model. The corresponding correlation loading plots (Fig. 8c and d) were used to identify the specific wavelengths contributing to this separation. For white eggs, the most influential wavelengths were identified in the ranges of 531–632 nm and 645–726 nm. For brown eggs, the key ranges were 535–655 nm and 684–747 nm, respectively. Notably, the high loadings in the 500-600 nm region are particularly significant, as this range corresponds to the maximum absorption peak of phosphatidylcholine (approximately 525 nm), which constitutes a major portion of the phospholipids in egg yolk (Kovacs-Nolan et al., 2005). This confirms that the spectral differences are directly related to the biochemical changes in a runny yolk.

##### Classification model development

Following feature selection for both defect types, four supervised classification algorithms were employed to build predictive models: Linear Discriminant Analysis (LDA), Quadratic Discriminant Analysis (QDA), Support Vector Machine with a linear kernel (SVM-linear), and SVM with a radial basis function kernel (SVM-RBF). To prevent overfitting and ensure robust generalizability, model performance was evaluated using a stratified 5-fold cross-validation strategy. To strictly avoid data leakage, the dataset was partitioned into training (80%) and validation (20%) subsets prior to any preprocessing or feature extraction. Dimensionality reduction and specific feature selection were independently executed within each training fold, ensuring the validation data remained entirely isolated from the model-building phase.

## Results and discussions

### Blood spot detection

#### Image-based detection

The performance of the image-based system was evaluated using both a predefined threshold and several machine learning classifiers, and the results are summarized in Table 1. The threshold-based method correctly identified 92.00% of the white eggs and 88.00% of the brown eggs with blood spots, while achieving 100.00% accuracy for non-defective eggs of both types. Among the machine learning models, Linear Discriminant Analysis (LDA) provided the best performance, achieving an accuracy of 88.00% and 84.00% for detecting blood spots in white and brown eggs, respectively. The slightly lower accuracy in brown eggs is likely due to the pigmentation of the shell, which has an absorption spectrum close to that of hemoglobin, making the defect less distinct (Fairchild, 2002; Liu et al., 2012). These results compare favorably with those of previous studies that used more complex systems, such as hyperspectral imaging and neural networks (K. (Hashemzadeh and Farajzadeh, 2016; K. Das & M. D. Evans, 1992; V. C. Patel et al., 1996).

**Table 1:** Classification accuracies for blood-spot detection using image-based methods.

Egg Type	Method	Blood Spot Egg	No Blood Egg
		Correct/Total (%)	Correct/Total (%)
White	Threshold (β=80)	92/100 (92.00%)	100/100 (100.00%)
	LDA	88/100 (88.00%)	96/100 (96.00%)
	QDA	84/100 (84.00%)	95/100 (95.00%)
	SVM-linear	85/100 (85.00%)	93/100 (93.00%)
	SVM-RBF	83/100 (83.00%)	91/100 (91.00%)
Brown	Threshold (β=80)	88/100 (88.00%)	100/100 (100.00%)
	LDA	84/100 (84.00%)	92/100 (92.00%)
	QDA	82/100 (82.00%)	91/100 (91.00%)
	SVM-linear	81/100 (81.00%)	92/100 (92.00%)
	SVM-RBF	83/100 (83.00%)	89/100 (89.00%)

The remarkable performance of the simple threshold method (β=80) in image-based blood spot detection, achieving 92.00% accuracy for white eggs and 88.00% for brown eggs with perfect specificity for normal eggs, can be attributed to the method's ability to exploit the natural binary contrast between blood spots and surrounding egg contents (Lawrence et al., 2008). The optimal threshold value effectively discriminates between dark, hemoglobin-rich blood spots and lighter egg material, leveraging the inherent optical properties differences that make threshold-based segmentation particularly suitable for this application (Mertens et al., 2006). The simplicity of this approach provides protection against overfitting common in agricultural machine learning applications with limited datasets, while maintaining computational efficiency crucial for real-time industrial egg grading systems (De Ketelaere et al., 2004).

#### Spectroscopy-based detection

The classification models developed using Vis-NIR spectroscopy demonstrated significantly higher accuracy for blood spot detection than image-based methods. The performance of the four classifiers for both the white and brown eggs is summarized in Table 2. The Quadratic Discriminant Analysis (QDA) model provided the best overall performance, achieving 97.50% accuracy for white eggs and 98.37% for brown eggs. This suggests that the relationship between the spectral data and the presence of blood spots is likely nonlinear, which QDA can model more effectively than linear methods. These results surpass the accuracy of previous studies that used SVM-based models or other spectroscopic methods (Chen et al., 2015; Feng et al., 2019).

**Table 2:** Overall accuracy of different classifiers for blood spot detection using spectroscopy.

Classification Model	Overall Accuracy (%)	
	White Egg	Brown Egg
LDA	89.00	96.75
QDA	97.50	98.37
SVM-linear	95.50	97.97
SVM-RBF	97.00	97.97

**Fig. 9:**
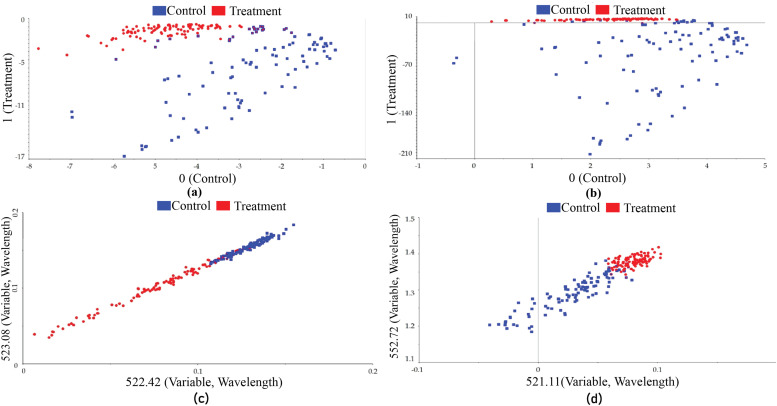
Classification plots for blood spot detection in white eggs. (a) LDA, (b) QDA, (c) SVM-linear, and (d) SVM-RBF.

The classification plots in Fig. 9 provide a visual confirmation of these results for the white eggs. The QDA plot (Fig. 9b) shows the clearest separation between the control (blue dots) and treatment (red dots) groups, with a curved decision boundary effectively isolating the two classes with minimal overlap. This clean visual separation justifies the high accuracy score. The SVM-RBF model (Fig. 9d) also performed well visually, consistent with its 97.00% accuracy. In contrast, the linear models, LDA (Fig. 9a) and SVM-Linear (Fig. 9c), show more mixing of the data points near their straight-line boundaries, which visually explains their lower accuracy scores of 89.00% and 95.50%, respectively.

**Fig. 10:**
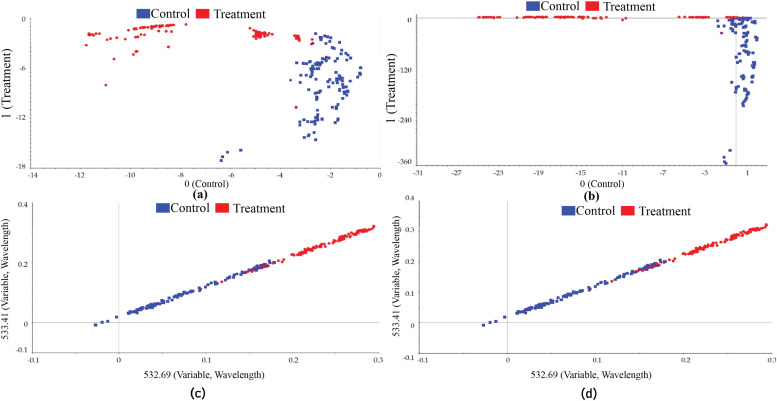
Classification plots for blood spot detection in Brown eggs. (a) LDA, (b) QDA, (c) SVM-linear, and (d) SVM-RBF.

A similar trend was observed for the brown eggs, as shown in Fig. 10. The QDA model (Fig. 10b) again provides the most distinct visual separation, which aligns with its highest accuracy of 98.37%. The SVM-RBF and SVM-linear models (Fig. 10d and c) also show strong performance with clear separation, justifying their high accuracies. The LDA model (Fig. 10a), while still performing well, showed slightly more overlap between the two groups, which is consistent with its having the lowest accuracy of the four models for brown eggs.

The consistent superiority of Quadratic Discriminant Analysis (QDA) over Linear Discriminant Analysis (LDA) across both spectroscopy applications reflects QDA's ability to model different covariance matrices for each class, better capturing the natural heteroscedasticity in spectroscopic data where blood spot and normal egg samples exhibit distinct variance patterns ( McLachlan, 2004). This flexibility allows QDA to accommodate the complex, non-linear relationships inherent in high-dimensional spectral signatures, where normal eggs show consistent patterns while defective eggs exhibit greater spectral diversity due to variations in defect characteristics (Hastie, 2009). The quadratic decision boundaries can better separate classes in spectroscopic applications, explaining QDA's superior performance of 97.50-98.37% compared to LDA's 89.00-96.75% accuracy rates (Bishop and Nasrabadi, 2006).

The performance differences between linear and radial basis function (RBF) Support Vector Machines reflect the fundamental trade-off between model complexity and generalization capability in spectroscopic applications (Vapnik, 2013). SVM-RBF's ability to model non-linear relationships through kernel transformations enables better capture of complex spectral interactions between wavelengths, which are common in biological samples where molecular absorption and scattering create intricate patterns (Cristianini and Shawe-Taylor, 2000). However, the RBF kernel's increased flexibility makes it more susceptible to overfitting with limited training data, explaining why performance improvements over linear SVM were sometimes marginal, requiring careful regularization parameter tuning to achieve optimal generalization (Schölkopf and Smola, 2002).

The substantial performance improvement when transitioning from image-based to spectroscopy-based methods reflects the fundamental advantage of accessing molecular-level information through visible and near-infrared light penetration (Nicolaï et al., 2007). Spectroscopic techniques can detect internal defects regardless of position within the egg while providing reduced interference from shell characteristics such as color variations and surface texture that commonly confound imaging systems (Hu et al., 2024). The high-dimensional spectroscopic data, typically containing hundreds of wavelengths, provides abundant quantitative features that are more reproducible and less susceptible to environmental variations compared to image-based measurements, enabling more robust classification models (Williams and Norris, 1987).

The differential performance between white and brown eggs reveals how shell pigmentation affects various sensing modalities, with brown eggshells presenting challenges for image-based systems due to melanin pigments that reduce optical contrast between internal defects and surrounding contents (Roberts, 2004). Conversely, spectroscopic methods often showed improved performance on brown eggs, suggesting that additional chromophores in brown shells may enhance spectral discrimination by creating more distinctive signatures between normal and defective eggs (Bamelis et al., 2008). The protein and mineral composition differences between shell types contribute to varying light penetration patterns and scattering characteristics that differentially affect detection algorithms (Ketelaere et al., 2002)

### Runny yolk detection

#### Image-based detection

As established in the methodology, the image processing techniques used for blood spot detection were not effective in identifying runny yolk. The diffuse nature of the defect did not present a distinct, segmentable region of interest, rendering the image-based approach unviable for this classification task.

#### Spectroscopy-based detection

This study presents the first successful non-destructive method for detecting runny yolk, which can be a significant advancement in the egg industry. The classification models developed using Vis-NIR spectroscopy achieved exceptionally high accuracy, as detailed in Table 3. The LDA model was the most effective, with an accuracy of 99.01% for white eggs. For brown eggs, all four classifiers achieved perfect 100.00% accuracy. This remarkable performance is likely due to the distinct and consistent spectral signature created by the diffusion of yolk into the albumen in the artificially induced defects. Spectral analysis leveraged the fact that egg yolk has a significantly higher absorption coefficient than albumen, particularly in the 500–600 nm range, which corresponds to the absorption peak of phosphatidylcholine, a major component of egg phospholipids (Kovacs-Nolan et al., 2005; Pickering, 1992). However, the distinct and highly classifiable spectral signature of a runny yolk extends beyond mere lipid absorption. The rupture of the vitelline membrane initiates a complex biochemical cascade, including rapid water migration from the water-rich albumen into the yolk. This osmotic shift induces localized protein denaturation and the breakdown of the internal protein matrix network (K. Wang et al., 2024). These structural deteriorations fundamentally alter the internal refractive index of the egg's contents, causing significant shifts in the optical scattering coefficient (Pickering, 1992). Because Vis-NIR transmission effectively captures both chemical absorption and physical scattering phenomena, the near-perfect classification accuracy achieved by the models is driven by this combined opto-chemical disruption- where altered light scattering profiles from denatured proteins amplify the baseline absorption signatures of the diffused lipids.

**Table 3:** Overall accuracy of different classifiers for runny yolk detection using spectroscopy.

Classification Model	Overall Accuracy (%)	
	White Egg	Brown Egg
LDA	99.01	100.00
QDA	98.37	100.00
SVM-RBF	99.01	100.00
SVM-Linear	99.01	100.00

b.

**Fig. 11:**
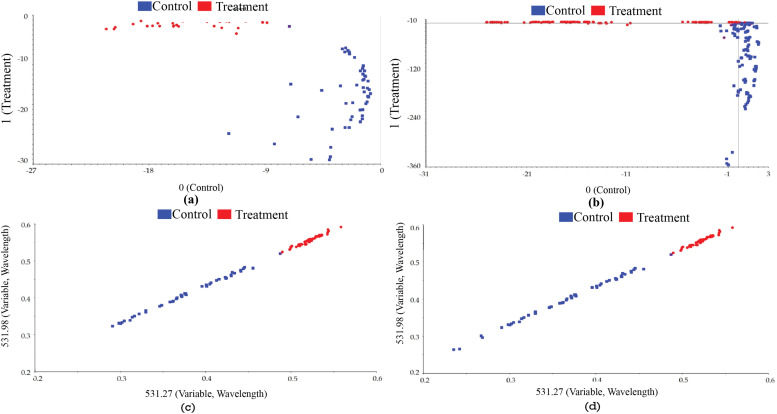
Classification plots for runny yolk detection in White eggs. (a) LDA, (b) QDA, (c) SVM-linear, and (d) SVM-RBF.

The classification plots for white eggs with runny yolk defects are presented in Fig. 11. The LDA plot (Fig. 11a) shows a clear and distinct separation between the control and treatment groups, which visually supports its high accuracy of 99.01%. The SVM-Linear and SVM-RBF models (Fig. 11c and d) also demonstrated excellent separation, consistent with their high accuracy scores. The QDA model (Fig. 11b), while still performing well, shows slightly more overlap between the two classes, which aligns with its slightly lower yet still high accuracy of 98.37%.

**Fig. 12:**
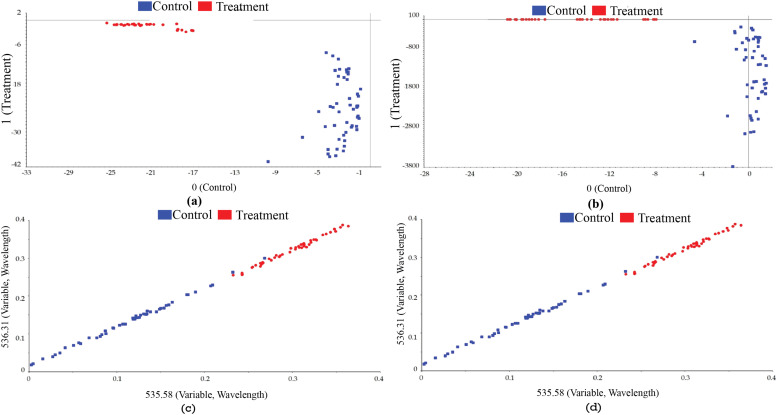
Classification plots for runny yolk detection in Brown eggs. (a) LDA, (b) QDA, (c) SVM-linear, and (d) SVM-RBF.

For brown eggs, the classification plots in Fig. 12 show a perfect separation for all four models, with no overlap between the control and treatment groups. This visual evidence directly corresponds to the 100.00% accuracy achieved by all classifiers for the brown eggs, as reported in Table 3.

The near-perfect accuracy achieved across all algorithms for runny yolk detection reflects the fundamental structural differences between normal gel-like yolks and defective liquid yolks, which create distinct optical scattering and absorption patterns easily detectable by spectroscopic methods (Li-Chan et al., 1995). The protein denaturation and network breakdown leading to runny yolk formation significantly alters molecular environments and water distribution, generating strong spectral signatures in the near-infrared region where water absorption bands are sensitive to hydrogen bonding patterns (Kemps et al., 2006). The binary nature of yolk consistency and the large volume fraction occupied by yolk within eggs provide strong, consistent signals that dominate spectroscopic measurements, making this defect inherently more amenable to detection regardless of the classification algorithm employed (Mertens et al., 2006).

#### Overall performance evaluation

While overall accuracy provides a general measure of performance, industrial applications require a more nuanced evaluation of false positives and false negatives. To this end, Precision, Recall (Sensitivity), and F1-scores were calculated for the optimal classification models (Table 4). Precision reflects the system's ability to avoid false positives (e.g., rejecting a healthy egg), while Recall measures the ability to detect all defective eggs.

**Table 4:** Detailed performance metrics for the optimal classification models (QDA for blood spots, LDA for runny yolk) compared to image-based thresholds.

Detection Method	Model	Egg Type	Accuracy (%)	Precision (%)	Recall (%)	F1-Score (%)
Blood Spot (Image)	Threshold	White	96.00	100.00	92.00	95.83
		Brown	94.00	100.00	88.00	93.62
Blood Spot (Spec)	QDA	White	97.50	100.00	95.00	97.44
		Brown	98.37	100.00	96.75	98.35
Runny Yolk (Spec)	LDA	White	99.01	100.00	98.02	99.00
		Brown	100.00	100.00	100.00	100.00

As shown in Table 4, the image-based thresholding method achieved 100.00% precision, indicating zero false positives. This is industrially significant as it ensures that no sound eggs are wasted. However, its Recall was lower (92.00% for white, 88.00% for brown), reflecting the limitation of imaging in detecting very small or subtle blood spots. In contrast, the spectroscopy-based models (QDA for blood spots and LDA for runny yolk) demonstrated a superior balance, achieving high scores across all metrics. Notably, the runny yolk detection models achieved perfect or near-perfect F1-scores (>99.00%), confirming that Vis-NIR spectroscopy is the most robust method for detecting internal diffusion defects that lack distinct visual boundaries.

#### Comparative analysis with existing detection technologies

To evaluate the industrial significance of the proposed system, it is essential to compare its performance against existing single-modality technologies, including manual candling, machine vision, and standalone spectroscopy. Because this foundational study utilizes an artificially induced dataset to guarantee ground-truth severity, a direct, horizontal comparison with experienced human candlers or commercial equipment on this specific batch was not conducted. Such a comparison would lack ecological validity, as operators and commercial algorithms are calibrated exclusively for naturally occurring defects. Consequently, the following sections compare the proposed method against established literature benchmarks to demonstrate theoretical feasibility.

##### Comparison with manual candling

The current industrial standard, manual candling, relies heavily on human visual acuity. Studies indicate that manual candling accuracy for internal defects typically ranges from 50% to 80%, heavily depending on operator fatigue and the throughput speed. Specifically, manual inspection often fails to detect small blood spots (<2 mm) or those obscured by the chalazae. Furthermore, diffused defects like runny yolk lack the distinct edges required for visual detection, often leading to a near-zero detection rate for this defect until the egg is broken. In contrast, the proposed Vis-NIR method achieved 99–100% accuracy for runny yolk, offering a reliable non-destructive alternative to the current destructive testing methods (Tang et al., 2024).

##### Comparison with computer vision systems

Computer vision (CV) systems have automated the candling process, significantly improving speed. Previous studies, such as those by V. C. Patel et al. (1996) and Pourreza et al. (2008), reported blood spot detection accuracies between 85% and 90%. However, these systems face a critical limitation: shell pigmentation interference. As observed in our study and supported by the literature, CV accuracy drops for brown eggs (from 92.00% to 88.00% in our results) because the melanin in the shell reduces the contrast between the blood spot and the background. Moreover, our results confirmed that CV is ineffective for runny yolk detection (0% accuracy) because the defect involves a chemical diffusion of lipids rather than a morphological object that casts a shadow.

##### Comparison with standalone spectroscopy

Spectroscopy has historically proven superior for assessing internal chemical properties, such as freshness (Haugh Unit). Our study validates this, showing that spectroscopy (QDA) outperformed imaging for blood spots (97.5–98.40% vs. 88–92%). This aligns with findings by (Chen et al., 2015), who reported high accuracy using Vis-NIR for blood spots. However, single-point spectroscopy can sometimes miss small, localized defects if the light path does not intersect the specific defect location. By integrating imaging (which scans the whole egg area) with spectroscopy, theoretically minimizes this spatial risk, although our results showed that spectroscopy alone was highly effective for the diffuse "runny yolk" defect due to the global change in albumen turbidity.

##### The Advantage of parallel modalities

The primary innovation of this study lies in the complementary nature of the evaluated technologies when applied in parallel. While imaging provides a rapid, spatial assessment effective for larger blood spots and shell integrity, it is blind to chemical defects. Spectroscopy fills this gap by detecting the molecular signatures of hemoglobin and lipids. Consequently, this **parallel** framework expands the application scope of automated grading systems, enabling the detection of a wider range of defects (both morphological and chemical) with a reliability that neither technology can achieve in isolation.

## Limitations and future scope

While this study demonstrates the high potential of integrating imaging and spectroscopy, it is critical to frame these findings as a foundational proof-of-concept. A primary limitation is the reliance on artificially induced defects to establish a controlled dataset. Artificial induction was strictly necessary for this initial stage to ensure precise knowledge of the defect type and severity (ground truth) for accurate model training. This level of control is exceptionally challenging with naturally occurring internal defects, particularly runny yolk, where non-destructive verification is impossible prior to breaking the egg.

While the artificial defects effectively mimic the targeted optical properties (hemoglobin absorption and lipid diffusion), they do not fully capture the variability of naturally occurring defects, which may exhibit greater diversity in size, position, viscosity, and concurrent abnormalities. Consequently, the current models' immediate generalizability to real-world industrial scenarios remains limited. To bridge the gap between this proof-of-concept and practical utility, our immediate future work will focus on validating and retraining these models using a large-scale dataset of naturally defective eggs sourced directly from commercial high-speed grading lines.

Additionally, this preliminary study focused exclusively on binary classification (the presence or absence of a defect) to establish baseline detectability. In commercial grading, systems must distinguish between "allowable" small blood spots and "rejectable" large blood spots. Reliably simulating a standardized severity gradient via artificial injection is highly prone to physical inconsistencies. Therefore, expanding the classification models to include multi-level severity grading is a primary objective for our subsequent studies involving naturally occurring defects.

Concurrently, this future natural-defect dataset will be utilized to benchmark more complex, supervised feature selection algorithms, such as Successive Projections Algorithm (SPA), Competitive Adaptive Reweighted Sampling (CARS), and Random Forest (RF), to further optimize model stability and computational efficiency for real-time grading.

Furthermore, transitioning these models to commercial grading lines will require addressing potential model drift caused by extreme physical variations in the eggs. Future system iterations will incorporate formal covariate analysis- integrating real-time physical parameters such as egg weight and shell thickness alongside the spectral data- to maintain classification robustness under highly variable, real-world conditions.

Furthermore, to intuitively demonstrate the superiority of the proposed framework, this future dataset of naturally occurring defects will be utilized to conduct a controlled, horizontal comparison against both experienced human candlers and existing commercial grading equipment under real-world operational speeds.

This foundational study was conducted under highly controlled laboratory conditions to isolate the baseline optical signatures of the defects. Real-world industrial environments introduce significant external noise, including ambient lighting fluctuations, surface eggshell stains (e.g., feces or dirt), and variations in egg storage time, all of which can alter transmission spectra and degrade model accuracy. Consequently, alongside testing naturally occurring defects, future research must incorporate rigorous robustness experiments to evaluate and optimize model stability under these typical industrial interfering conditions.

Furthermore, while this study evaluated the imaging and spectroscopic systems independently, future iterations will explore true decision-level data fusion (e.g., ensemble voting or stacking algorithms) to mathematically integrate these data streams and potentially maximize overall classification accuracy.

## Conclusion

In this study, we successfully developed and validated a system for the non-destructive detection of internal egg defects. While the imaging system provided a reliable method for detecting blood spots, Vis-NIR spectroscopy was more accurate and uniquely capable of detecting runny yolk. The high accuracy of the spectroscopic method, particularly for runny yolk detection, represents a significant advancement in the assessment of egg quality. The findings of this study provide a strong foundation for the development of automated, high-throughput egg grading systems that can improve efficiency, reduce waste, and enhance consumer confidence in the poultry industry. Future studies should focus on validating these methods with naturally occurring defects and exploring the use of alternative light sources, such as LEDs, to improve performance in the NIR range.

## Declaration of generative AI and AI-assisted technologies in the writing process

During the preparation of this work, the authors refined the language using Gemini AI and Paperpal. After using this service, the authors reviewed and edited the content as needed and take full responsibility for the content of the published article.

## CRediT authorship contribution statement

**Abdullah Al-Mamun:** Writing – original draft, Visualization, Software, Methodology, Formal analysis, Data curation, Conceptualization. **Md. Abdullah Al Noman:** Writing – original draft, Visualization, Software, Methodology, Formal analysis, Data curation, Conceptualization. **Iqbal Hossain:** Writing – original draft, Visualization, Software, Investigation, Formal analysis, Data curation. **Jannatul Ferdushi Jany:** Writing – review & editing, Validation, Methodology, Investigation. **Md. Anisur Rahman Mazumder:** Writing – review & editing, Validation, Resources, Project administration, Methodology. **Abdullah Iqbal:** Writing – review & editing, Validation, Supervision, Project administration. **Mohammad Gulzarul Aziz:** Writing – review & editing, Supervision, Resources. **Afzal Rahman:** Writing – review & editing, Validation, Supervision, Resources, Project administration, Methodology, Investigation, Conceptualization.

## Disclosures

The authors declare that they have no known competing financial interests or personal relationships that could have appeared to influence the work reported in this paper.
